# Caldesmon regulates the motility of vascular smooth muscle cells by modulating the actin cytoskeleton stability

**DOI:** 10.1186/1423-0127-17-6

**Published:** 2010-02-03

**Authors:** Qifeng Jiang, Renjian Huang, Shaoxi Cai, Chih-Lueh A Wang

**Affiliations:** 1 Key Laboratory of Biorheological Science and Technology, Ministry of Education, Bioengineering College, Chongqing University, Chongqing, 400044, China; 2 Boston Biomedical Research Institute, 64 Grove St, Watertown, MA 02472, USA

## Abstract

**Background:**

Migration of vascular smooth muscle cells (SMCs) from the media to intima constitutes a critical step in the development of proliferative vascular diseases. To elucidate the regulatory mechanism of vacular SMC motility, the roles of caldesmon (CaD) and its phosphorylation were investigated.

**Methods:**

We have performed Transwell migration assays, immunofluorescence microscopy, traction microscopy and cell rounding assays using A7r5 cells transfected with EGFP (control), EGFP-wtCaD or phosphomimetic CaD mutants, including EGFP-A1A2 (the two PAK sites Ser452 and Ser482 converted to Ala), EGFP-A3A4 (the two Erk sites Ser497 and Ser527 converted to Ala), EGFP-A1234 (both PAK- and Erk-sites converted to Ala) and EGFP-D1234 (both PAK- and Erk-sites converted to Asp).

**Results:**

We found that cells transfected with wtCaD, A1A2 or A3A4 mutants of CaD migrated at a rate approximately 50% more slowly than those EGFP-transfected cells. The migration activity for A1234 cells was only about 13% of control cells. Thus it seems both MAPK and PAK contribute to the motility of A7r5 cells and the effects are comparable and additive. The A1234 mutant also gave rise to highest strain energy and lowest rate of cell rounding. The migratory and contractile properties of these cells are consistent with stabilized actin cytoskeletal structures. Indeed, the A1234 mutant cells exhibited most robust stress fibers, whereas cells transfected with wtCaD or A3A4 (and A1A2) had moderately reinforced actin cytoskeleton. The control cells (transfected with EGFP alone) exhibited actin cytoskeleton that was similar to that in untransfected cells, and also migrated at about the same speed as the untransfected cells.

**Conclusions:**

These results suggest that both the expression level and the level of MAPK- and/or PAK-mediated phosphorylation of CaD play key roles in regulating the cell motility by modulating the actin cytoskeleton stability in dedifferentiated vascular SMCs such as A7r5.

## Background

Migration of vascular smooth muscle cells (SMCs) from media to intima is a critical step in the development of proliferative vascular diseases such as atherosclerosis, and in response to vascular injuries such as angioplasty and organ transplatation. Fully differentiated SMCs normally do not proliferate nor migrate. Upon stimulation, however, SMCs can dedifferentiate and change from contractile to synthetic phenotypes, which enable cell proliferation and migration. During this process SMCs undergo cellular remodelling and a number of smooth muscle-specific contractile proteins are converted to non-muscle isoforms. One of such signature proteins is caldesmon (CaD).

CaD is an actin-binding protein that also interacts with myosin, tropomyosin and calmodulin [1]. The two alternatively spliced isoforms of CaD derive from a single gene [2]: the heavy caldesmon (h-CaD), found exclusively in differentiated SMCs, and the light isoform (l-CaD), present in nearly all types of vertebrate cells. Unlike visceral smooth muscles, which only express h-CaD, vascular smooth muscles contain both h- (>75%) and l-CaD (<25%) [3]. However, upon dedifferentiation, h-CaD is rapidly degraded in vascular SMCs, and only l-CaD is expressed. Therefore, l-CaD is the form that is closely associated with the synthetic type of smooth muscle organs.

Both h- and l-CaD bind actin filaments and stabilize the filamentous structure. In SMCs h-CaD, together with tropomyosin, modulates the actomyosin ATPase activity by reversibly and cooperatively inhibiting myosin binding to actin [4]. The inhibitory effect of h-CaD on muscle contractility has been demonstrated by peptide intervention [5-8] and antisense knockdown [9] experiments. Reversal of this inhibition is accompanied by phosphorylation of h-CaD at MAPK-specific sites [10], which partially dissociates h-CaD from actin filaments and allows myosin to bind [11]. The C-terminal region of h-CaD can also be phosphorylated by PAK [12] in vitro, although it is less clear whether or not the in vivo modification occurs in SMCs.

In non-muscle cells l-CaD appears to have more diverse functions. l-CaD has been reported to be involved in cell division [13], migration [14], adhesion [15], postmitotic spreading [16], apoptosis [17], and intracellular granule movement [18]. Like that of h-CaD, the action of l-CaD is also regulated via MAPK-mediated phosphorylation by such enzymes as cdc2 kinase [13] and Erk1/2 [14]. We have previously shown that, when cells are stimulated with phorbol ester, l-CaD is phosphorylated at the Erk sites and moves from stress fibers in the cytosol to nascent focal contacts at cell peripheries [16]. Phosphorylation of l-CaD by PAK also affects the morphology and migratory properties of non-muscle cells [19]. Consistently, l-CaD is present in podosomes [20,21], where it regulates podosome dynamics in a PAK-dependent manner [22].

The fact that the presumed functions of CaD are affected by MAPK and PAK has inspired much interest. Both types of kinases add phosphate groups to residues near the actin-binding sites at the C-terminal region of CaD, thereby decreasing its effectiveness of actin binding, as well as its stabilizing action on the actin cytoskeleton. This may suggest that CaD (particularly l-CaD) serves as a converging point for the MAPK and PAK signaling under the stimulation of a wide variety of agonists; it also raises the question as how the two types of CaD phosphorylation relate to each other. In this work we have analyzed the cellular consequences of the MAPK and PAK actions, individually and in combination, on l-CaD in a dedifferentiated SMC line (A7r5). By using phosphomimetic mutagenesis, we aimed to dissect the effect of MAPK- and PAK-mediated phosphorylation on CaD. Our results indicate that CaD phosphorylation is an obligatory step for cell motility, and that MAPK and PAK work independently and additively toward this process. Since only l-CaD is expressed in these cultured cells, CaD refers to the non-muscle isoform exclusively throughout this work except otherwise specified.

## Methods

### Cell Culture

Rat aorta smooth muscle cells A7r5 (ATCC# CRL-1444™) were maintained in DMEM (Cellgro™) supplemented with 10% fetal bovine serum (Cellgro™) and 1% antibiotics (Penicillin-Streptomycin, Cellgro™). Cells were cultured at 37°C under a 5% CO_2 _atmosphere.

### Plasmids

The pCB6 hx plasmid containing the cDNA of human l-CaD (GeneBank #M64110) was originally a gift from Dr. Jim Lin (University of Iowa, Iowa City, IA). The insert was subcloned into the mammalian expression vector pEGFPC1 (Clontech). Site-directed mutagenesis was performed as previously described [16] with the two PAK sites Ser452 and Ser482 converted to Ala (EGFP-A1A2); the two Erk sites Ser497 and Ser527 to Ala (EGFP-A3A4); both PAK- and Erk-sites to Ala (EGFP-A1234); or both PAK- and Erk-sites to Asp (EGFP-D1234).

### Cell Transfection

A7r5 cells were plated at 60% confluence in 6-well cell culture plates. 18 h after plating, the cells were starved for 2 h before transfection using the Lipofectamine™ reagent supplemented with Plus™ reagent (Invitrogen). Briefly, 2 μg DNA in 5 μl Plus™ reagent and in 4 μl Lipofectamine™ reagent were diluted, respectively, with 43 μl and 46 μl serum-free DMEM medium. After 20 min incubation at room temperature, the two solutions were mixed and incubated for another 20 min. The mixture was then added to the cells; after 5 h the transfection medium was replaced with full medium.

### Western Blot Analysis

The expression level of exogenous CaD induced by transfection, and the extent of CaD phosphorylation in A7r5 cells were evaluated by Western blot analysis using a Odyssey Infrared Imaging System by Li-COR Biosciences (Lincoln, NE) [23,24] as described previously [16]. Cells transfected with vehicle alone (EGFP), EGFP-wtCaD (wtCaD), EGFP-A1A2 (A1A2), EGFP-A3A4 (A3A4), EGFP-A1234 (A1234), or EGFP-D1234 (D1234) were seeded at 10^5 ^cells/well on 6-well cell culture plates (Becton-Dickinson, Rutherford, NJ). After 24 h incubation, culture medium was removed, and cells were rinsed twice with ice-cold PBS. Proteins were extracted by adding to each well 150 μl of lysing buffer containing phenylmethylsulfonyl fluoride 1 mM (Sigma), leupeptin 10 mg/ml (Sigma), aprotinin 30 mg/ml (Sigma), and NaVO_3 _1 mM (Sigma). The plates were incubated on ice for 30 min and scraped. Total cell extracts were separated on SDS-PAGE and immunoblotted with lab-made polyclonal anti-CaD and affinity purified polyclonal anti-pSer527 (Ser527 of l-CaD is equivalent to Ser789 in h-CaD), as well as monoclonal anti-β-actin (Sigma), followed by affinity purified anti-rabbit and anti-mouse secondary antibodies conjugated with IRDyeTM 700 and 800, respectively. The digitized fluorescent bands were integrated, and the ratios (GFP-tagged CaD to endogenous CaD) were calculated for both protein level and phosphorylation of each pair after normalized against the amount of β-actin, which was used as a loading reference.

### Fluorescence Microscopic Imaging

For fluorescence microscopy, cells transfected with EGFP, wtCaD, A1A2, A3A4, A1234, or D1234 were seeded on glass coverslips placed in a plastic culture dish and incubated overnight, during which time the cell became well-spread. Cells were then starved for 24 h and washed in PBS, fixed for 15 min in freshly prepared 4% paraformaldehyde (PFA) in PBS and permeabilized with 0.3% Triton X-100 in 4% PFA in PBS for 5 min. For all subsequent steps, solutions were prepared in PBS. Cells were thoroughly rinsed in PBS between steps and incubations were performed at room temperature. F-actin was stained with rhodamine-phalloidin and incubated for 1 h. Finally, cell-loaded coverslips were rinsed and mounted on glass slides in Mowiol (Sigma). Images were obtained using a laser scanning system BioRad Radiance 2000 equipped with the confocal head attached to the Nikon Eclipse TE300 microscope. Data were acquired and analyzed with Laser Sharp 2000 BioRad software. All images were collected through single-section acquisition with scan performed from the top to the bottom of the cell in two-color (green and red) channels in parallel.

### Cell Migration Assay

Cell migration was assayed with 24-well tissue culture Transwell (Becton Dickinson) plates comprising a polycarbonate membrane with 8-μm pores. The inner and outer chamber membranes were coated with 5 μg/ml of human fibronectin (R&D, Minneapolis, MN) at 37°C for 2 h, and then rinsed with PBS. A7r5 cells transfected with EGFP, wtCaD, A1A2, A3A4, A1234, or D1234 were then seeded on the inner chamber of the Transwell plate at a concentration of 2 × 10^4 ^cells/well in 200 μl serum-free DMEM. The outer chamber was filled with 800 μl full culture medium which contained 10% FBS and 50 ng/ml FGF (R&D, Minneapolis, MN), and incubated for 36 h at 37°C. The number of total migrated cells and green (i.e., transfected) cells were counted in fields randomly chosen from 9 equally divided zones of the membrane in triplicates under phase-contrast and fluorescence channel with the ZEISS-AXIO fluorescence microscope system. The percentage of transfected cells in the total migrated cells was determined for each experiment. These numbers were then divided by the transfection efficiency to obtain the motility of the transfected cells relative to the untransfected cells.

### Fourier-Transform Traction Microscopy

A7r5 cells transfected with EGFP, wtCaD, A1A2, A3A4, A1234, or D1234 were seeded on the collagen-coated, fluorescence mircobeads-embedded polyacrylamide gel, which was prepared according to a previously described protocol [25,26], with the cell density kept lower than 10^4 ^per dish. After 24 h incubation, the cells were starved overnight prior to measurements. With fluorescence channel first followed by phase-contrast, the image of a single green fluorescence cell and that of the fluorescent micropatterned beads were recorded before and after trypsinization. The two images of the micropatterned beads plus the phase-contrast cell image were taken to calculate the displacement field of the gel generated by the cell [27]. The projected cell area was also calculated based on the cell contour determined from the phase-contrast image obtained at the start of the experiment. From the displacement field the traction field within a 50 μm × 50 μm square was calculated as described by Butler et al. [28]. The magnitude and the direction of the vectors corresponding to the traction imposed on the gel underneath the cell yielded a scalar measure of cell contractility (strain energy), which is the total energy (in pJ) transferred from the cell to the substratum.

### Cell Rounding Assays

Cell rounding assays after treatment with trypsin were performed as previously described [16].

### Statistics

All measurements are expressed in terms of mean ± standard deviation (SD), except those of calculated total strain energy, which are expressed in mean ± standard error (SE). Comparisons between 2 samples of unequal variance were performed by Student's *t*-test using 2-tailed distribution. *P *< 0.05 was considered as significant.

## Results

### Effect of phosphomimetic mutation of CaD on the morphology of A7r5 cells

The inhibitory action of CaD on the actomyosin interaction is known to be regulated by MAPK- [11] and PAK- [12,19] mediated phosphorylation. To probe the significance of such regulation, to dissect the effect of the two types of phosphorylation, and to test their combined effect on the actin cytoskeleton and motility of SMCs, we have designed EGFP-tagged phosphomimetic mutants of CaD and force-expressed them in A7r5 cells. Serine residues at positions 452 (designated as position #1) and 482 (position #2) are taken as the "PAK-sites", whereas serines at positions 492 (position #3) and 527 (position #4) are taken as the "Erk-sites", which may also be phosphorylated by other MAPKs. Mutants include A1A2 (PAK-sites disabled), A3A4 (Erk-sites disabled), A1234 (both PAK- and Erk-sites disabled) and D1234 (both PAK- and Erk-sites phosphorylated). Cells were also transfected with either EGFP-tagged wild-type CaD (wtCaD) or the vehicle alone (EGFP), the latter being used as controls. The cell viability was not appreciably affected by transient transfection. No sign of cell death was detected within the time period of experimentation.

**Figure 1:**
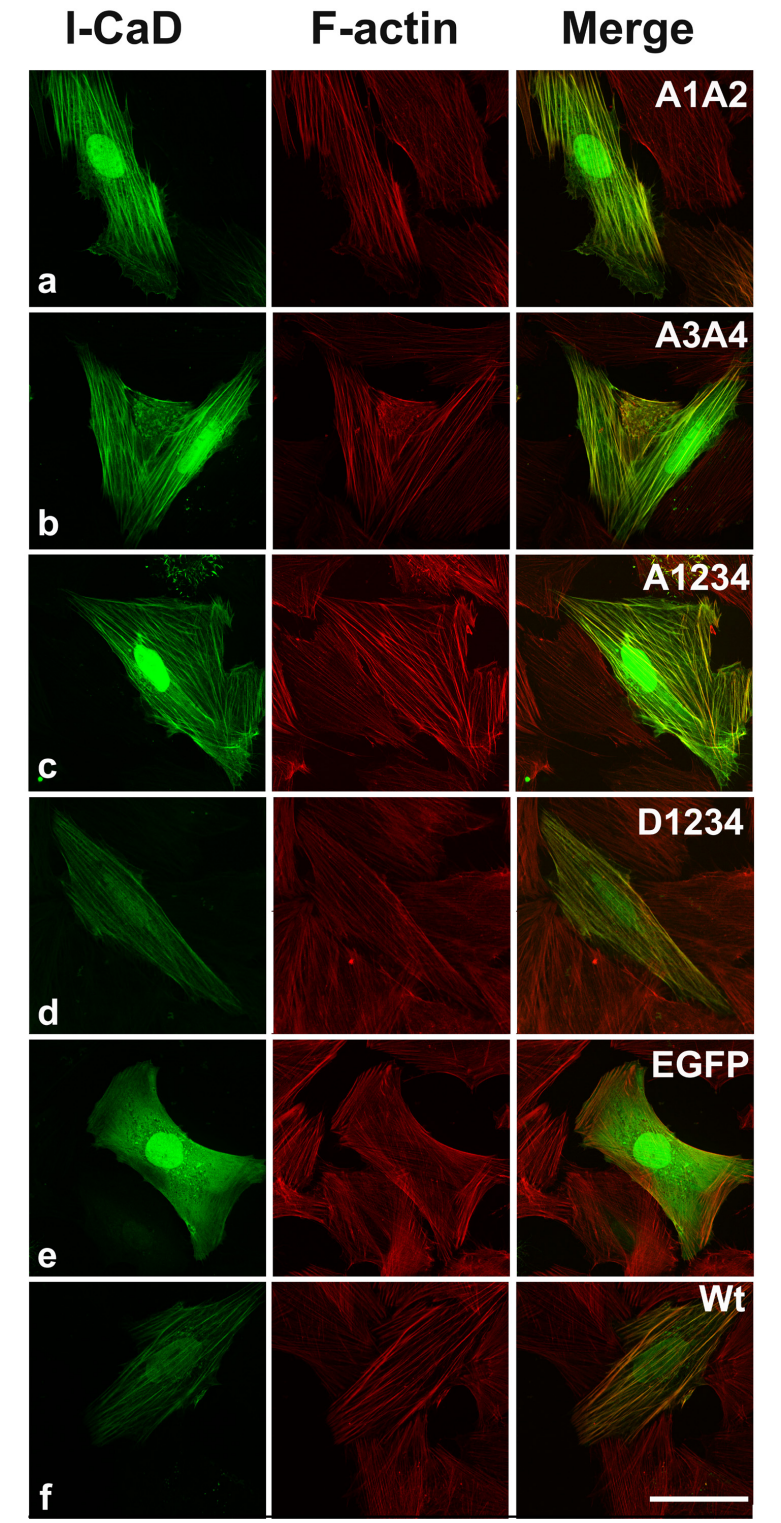
**Fluorescence images of transfected A7r5 cells**. All CaD constructs were EGFP-tagged (left panels); actin was stained with red (middle panels). Merged images are shown on the right panels. Cells were transfected with EGFP (control, Row e), EGFP-wtCaD (wild-type CaD; Row f) and CaD mutant, including EGFP-A1A2 (Row a), EGFP-A3A4 (Row b), EGFP-A1234 (Row c) and EGFP-D1234 (Row d). A1234 transfected cells had most robust cytoskeleton structure, the wtCaD, A1A2 and A3A4 transfected cells also had more robust structure than D1234 and control cells (EGFP). The D1234 transfected cells exhibited similar cytoskeleton structure to the control cells. Scale bar, 100 μm.

Among all constructs cells transfected with the A1234 mutant showed most robust cytoskeleton structure. The majority of A1234-expressing cells exhibited thicker and longer stress fibers than the untrasnfected cells and the EGFP-expressing control cells. The green fluorescence in these cells overlapped closely with the red phalloidin staining (Fig. 1-c), indicating that the expressed CaD mutant binds to the actin cytoskeleton with augmented stability. The wtCaD, A1A2 and A3A4 transfected cells also showed prominent cytoskeleton structures with similar overlapping distribution of EGFP-tagged CaD with stress fibers (Fig. 1-a,b,f), but the difference between transfected cells and untransfected cells was less striking than that for A1234. In contrast, the D1234-transfected cells exhibited much weaker stress fiber staining than cells transfected with the A-mutants and wt-CaD. Although some faint stress fibers were nevertheless detected in D1234-transfected cells, the exogenous CaD primarily overlapped with the F-actin staining at the cortical regions. Notably, the actin cytoskeleton in these cells exhibited little or no difference from that in the untransfected cells, as seen in the control cells (Fig. 1-d,e).

### Effect of phosphomimetic mutation of CaD on the motility of A7r5 cells

**Figure 2:**
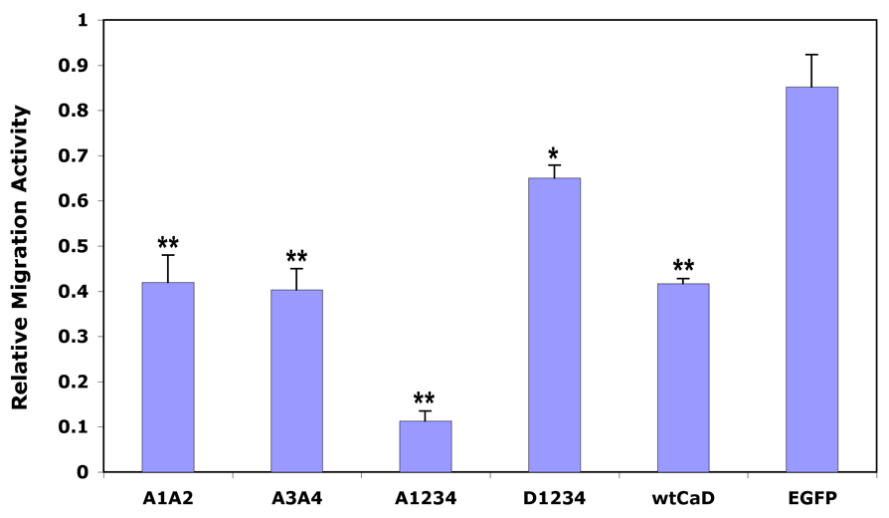
**Summary of Transwell migration assays**. Cells transfected with EGFP (control), EGFP-tagged wtCaD, A1A2, A3A4, A1234 and D1234 were subjected to migration assays, and the migration activity (see Methods) was compared to that of untransfected cells. The relative migration activity for the A1234-transfected cells was about 13% of the control cells (EGFP), and the ratios of A1A2 and A3A4 were 49% and 47% of the control cells, respectively. It seems that both Erk and PAK contributed equally to the motility of these two types of cells and this effects are additive. The D1234 mutant had a relatively weak inhibitory effect on the motility of A7r5 cells. The error bars represent the standard deviations (SD) of 3 independent measurements. Single (*) and double (**) asterisks on the peaks denote *P *< 0.05 and *P *< 0.005, respectively.

To determine the effect of phosphomimetic mutation of CaD on the motility of A7r5 cells, we have performed Boyden chamber migration assays using Transwell filters of 8-μm pore size. Cells were stimulated by 50 ng/ml FGF and 10% serum to undergo chemotactic migration. The motility of cells expressing different CaD mutants relative to the normal, untransfected cells was evaluated after 36 hours. Among all constructs the A1234 mutant resulted in most hindered motility. Taking untransfected A7r5 cells as a standard, we found that the relative migration activity for the A1234-transfected cells was only 0.113 ± 0.02 (n = 3; same below), which was about 13% of the EGFP-transfected control cells (0.85 ± 0.07; Fig. 2). Transfection with wtCaD also slowed down the rate of cell migration, but to a lesser extent (0.417 ± 0.01 of the control cells). The motility of D1234 transfected cells (0.65 ± 0.028) was higher than the cells transfected with all other CaD variants, but still about 24% lower than the control cells. It is clear that CaD plays an important role in controlling the activity of migration in A7r5 cells, and phosphorylation of CaD appears to facilitate this activity. Interestingly, cells transfected with A1A2 or A3A4 mutant of CaD migrated at a rate (0.42 ± 0.06 and 0.40 ± 0.04, respectively) that is approximately half way between the A1234 cells and the control cells. Apparently both MAPK and PAK contribute to the motility of A7r5 cells and these effects are comparable and additive.

### Effect of phosphomimetic mutation of CaD on the contractility of A7r5 cells

**Figure 3:**
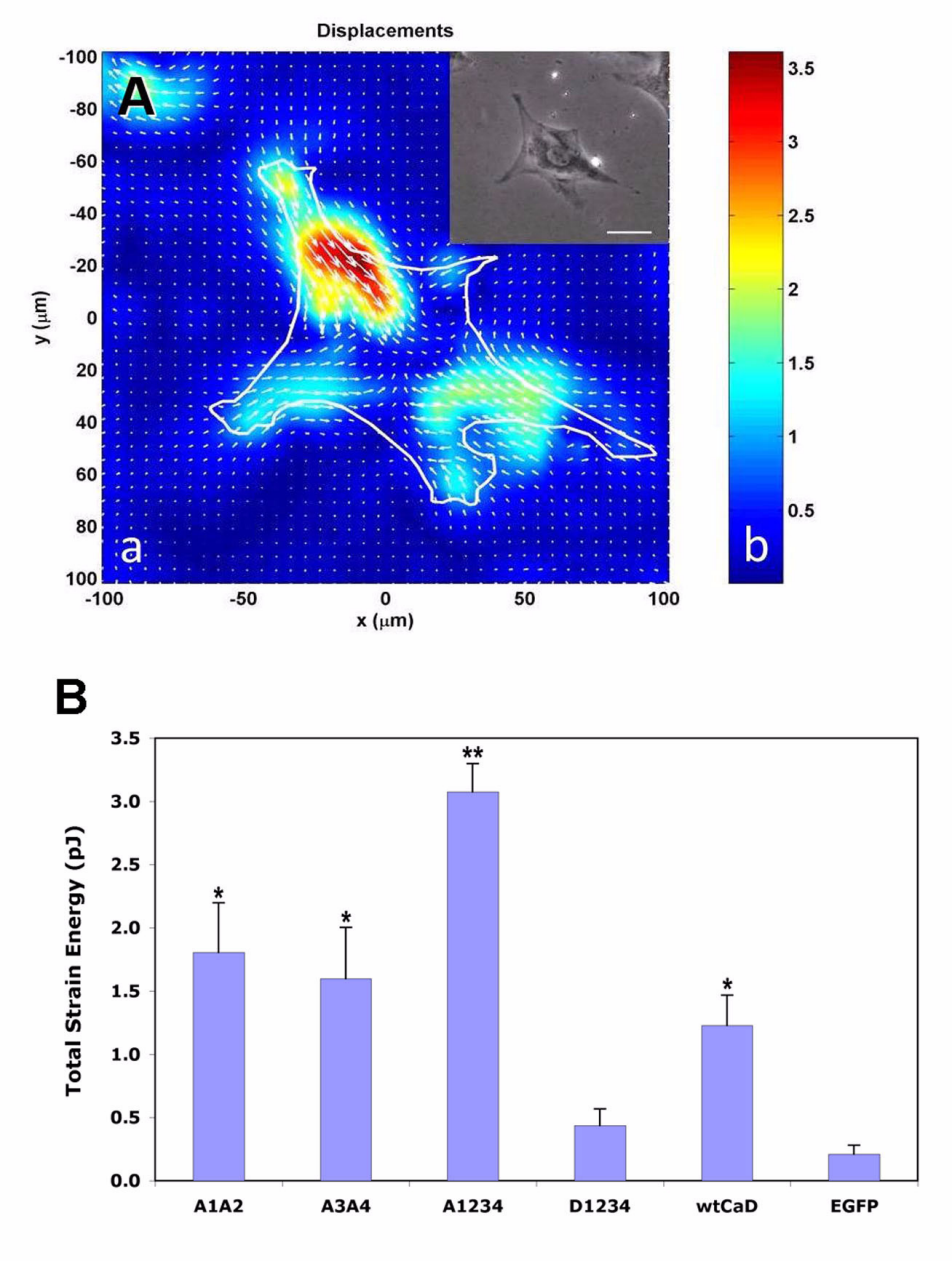
**(A) Fourier transform tranction microscopy measurements and the images of a representative A7r5 cell gathered in the measurement process**. (**a**) The single green cell was first identified under fluorescence channel and then the phase-contrast image (upper right) was recorded. Based on beads movement, the traction field was calculated by MATLAB. The magnitude and the direction of the vectors indicate the bead movement, which was used to compute the contractile moment. Scale bar: 50 μm. (**b**) The color coding for the magnitude of the bead movement. (**B**) Results of total strain energy measurements (in pJ) of A7r5 cells transfected with phosphomimetic mutants of CaD and EGFP alone (control). The error bars represent the standard errors (SE) of 10 measurements. Single (*) and double (**) asterisks on the peaks denote *P *< 0.05 and *P *< 0.005, respectively.

One of the key steps during cell migration is contraction by which the cell body is moved forward [29]. The observation that the A1234 mutant of CaD slowed down the overall rate of migration might lead to the prediction that the cell contractility is also hampered. To test whether this is the case and to decipher the origin of the observed effect mechanistically, we wished to determine how phosphomimetic mutations of CaD affect the contractility of A7r5 cells. Fourier-transform traction microscopy at the single cell level was used for this purpose. We have performed contractility assays using A7r5 cells transfected with EGFP, wtCaD, A1A2, A3A4, A1234 and D1234. Stationary, individual cells were first plated on the fluorescent microbeads-imbedded polyacrylamide gel slab and examined by fluorescence microscopy, while the cell images were recorded before and after trypsinization. From these images the displacement field of the fluorescent beads was calculated (Fig. 3A-a). The magnitude and the direction of the vectors corresponding to the bead movement underneath the cell were then used to compute the average strain energy (i.e., the energy that the cell transfers to the substratum owing to the contractile activity; in pJ) of the cell.

Contrary to our expectation, we found that the A1234 mutant transfected A7r5 cells that showed severely hampered motility (see Fig. 2), exhibited most strengthened, instead of compromised, contractility, among all CaD variant transfected cells (Fig. 3B). The A1234 mutant (3.08 ± 0.22 pJ) showed about 15-fold enhancement in the traction force measurement compared to the control cells (0.21 ± 0.07 pJ). Both the A1A2 (1.81 ± 0.39 pJ) and A3A4 (1.60 ± 0.40 pJ) mutants resulted in about 8-fold enhancement in total strain energy. The wtCaD transfected cells also had significantly higher traction force than the control cells, although not as high as the A1234 mutant transfected cells. The increases in the contractility caused by the wtCaD transfection were about 6-fold (1.23 ± 0.24 pJ). The D1234 mutant transfection showed little enhancement for cell contractility, the measured force for the D1234 mutant transfected cells (0.44 ± 0.13 pJ) was almost the same as that of the control cells. It should be mentioned that because the traction measurements were based on single cell experiments, the scattering of the data was relatively large even with multiple measurements for each set (n = 10). Nevertheless, from the trend of the data it is rather striking that the effect of phosphomimetic CaD transfection on the contractility is precisely reciprocal to that on the migratory activity: The cells with the highest migratory activity (i.e., the EGFP-expressing cells) showed the lowest strain energy, whereas the cells with the slowest migration (i.e., the A1234-expressing cells) had the strongest strain energy. Like in the case of migration assays, the PAK- (A1A2) or Erk- (A3A4) sites disabled mutants exhibited about 50% of the change displayed by the mutant with both types of phosphorylation sites disabled (A1234). Phosphorylation of CaD therefore must have similar but opposite effects on these two events. Another important finding from these data is that, since the strain energy results from actin-based contractile force, the observed lower migration activity of mutated A7r5 cells clearly was not owing to inhibited contractility.

### State of phosphorylation of CaD mutants by Western blot analysis

**Figure 4:**
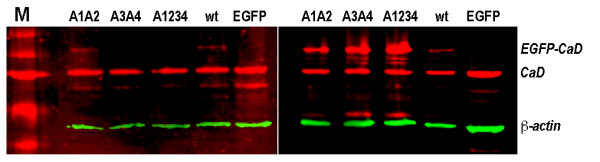
**Western blot analysis of CaD phosphorylation in the transfected A7r5 cells**. The total cell extracts from A7r5 cells transfected with various constructs were immunoblotted with polyclonal anti-pSer527 (Left Panel) and anti-CaD (Right Panel) antibodies. M: Molecular weight markers; samples (Lanes 2-6) are, respectively, cells transfected with A1A2, A3A4, A1234, wtCaD, and EGFP alone (Control). The corresponding ratios of the digitized intensity of the EGFP-tagged CaD variant (the upper red band; Right Panel) to that of the endogenous CaD (the lower red band; Right Panel) are, respectively, 0.93, 1.71, 2.60, 0.43 and, 0 (EGFP); and the corresponding ratios of the digitized intensity of the phospho-EGFP-CaD variant (the upper red band; Left Panel) to that of the phosphorylated endogenous CaD (the lower red band; Left Panel) are, respectively, 0.13 (A1A2), 0, 0, 0.10 (wtCaD) and, 0. The apparent lower signal in the phosphorylation for the engineered variants (human) than the endogenous CaD (rat) may be partly due to different immuno-reactivities of the antibodies. Moreover, since the transfection efficiency is ~35% in all cases, the actual ratios could be higher.

The fact that cells expressing the A1A2 and the A3A4 mutants exhibited about 50% of the overall changes found for the A1234 cells in both migration and contractile activities compared to those of the control cells implies: (a) PAK- and MAPK-mediated phosphorylation of CaD contributes equally and additively to these activities; and (b) the A1A2 and the A3A4 mutants might in fact be phosphorylated at *other *available sites in these cells. To test the latter idea, we have examined the phosphorylation status of the engineered CaD mutants by Western blot analysis. A lab-prepared polyclonal antibody against the Erk-site pSer527 (or pSer789 in h-CaD) was used for this purpose. Indeed, as shown in Fig. 4, the EGFP-tagged A1A2 and wtCaD, but not the A3A4 and A1234, were found positive for MAPK-mediated phosphorylation. Not surprisingly, the endogenous CaD in all cell lines was also phosphorylated at this Erk-site. Since we don't have the anti-PAK-sites antibody, a similar experiment to verify the PAK-mediated phosphorylation on the EGFP-tagged CaD variants is not possible at this time.

### Effect of phosphomimetic mutation of CaD on the detachment behavior of A7r5 cells upon trypsinization

**Figure 5:**
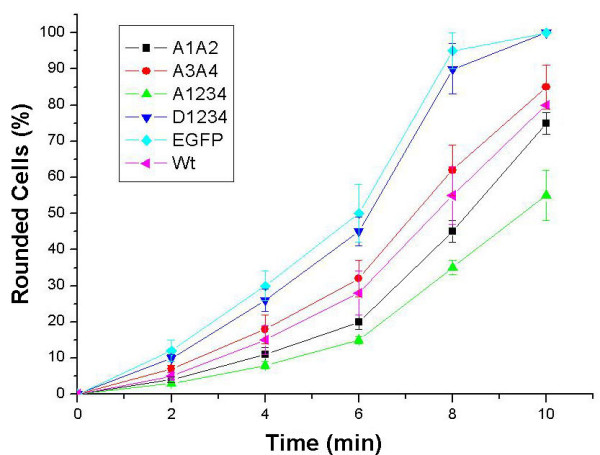
**Detachment of transfected A7r5 cells upon trypsinization**. A1A2- (squares), A3A4- (circles), A1234- (triangles), D1234- (inverse triangles), EGFP- (diamonds), wtCaD- (turned triangles) transfected cells were plated on 60 mm dishes. Cells from each plate were trypsinized and monitored under the phase-contrast and fluorescence microscope for time-dependent retraction, rounding and detachment. Percentages of round cells at 2, 4, 6, 8 and 10 min were plotted for each type of cells. Each point was an average of 6 independent measurements; error bars represent standard deviations.

Finally, in search of the cause for the decreased migration activity, we wished to test whether CaD mutation affects cell detachment, which constitutes another step critical to cell migration. Cells undergo rapid retraction and rounding, and eventual detachment from the substratum upon trypsin treatment because of disengagement of focal adhesions and partial disassembly of actin bundles. We used a simple assay by quantifying the number of rounded cells (including detached cells) as a function of time following trypsinization to compare the detachment kinetics of A7r5 cells transfected with either EGFP or CaD mutants. We found that cells transfected with wtCaD, A1A2 or A3A4 all showed delayed responses to trypsin digestion as compared to the control cells (Fig. 5). Even more hampered rounding was observed for the A1234 transfected cells, in agreement with previous observations with rat aortic fibroblast cells [16]. In contrast, the D1234 transfected cells showed similar kinetics of rounding up as the control cells. The detachment behavior thus parallels the migration activity.

## Discussion

CaD is known to bind actin and stabilize the filamentous structure. Binding of CaD to actin also inhibits the actomyosin interaction, and results in inhibition on many cellular processes such as migration, adhesion and proliferation [30]. These inhibitory actions can be reversed by binding to calmodulin in the presence of Ca^2+^, although it is more likely that the in vivo function of CaD is regulated by phosphorylation. In vivo CaD phosphorylation was documented not only in activated SMCs [31], but also in mitotic cells [32] and migrating smooth muscle [14] or non-muscle cells [16]. Because of these properties, CaD serves as a target for manipulation of cellular behaviors. There have been plenty of data in the literature using ectopic expression of CaD or mutants to probe cell movement; however, the results are not always consistent [16,17,20,33-38]. While most studies found over-expressed CaD stabilizes stress fibers in the cell and inhibits cell motility, one report [37] showed opposite results, in which case transient transfection of CaD not only disrupted stress fibers, but also disassembled focal adhesions. Because of this controversy, the exact function of CaD has not been settled, although the critical involvement of CaD in cell motility is widely recognized.

Notably, in that earlier study the phosphorylation status of CaD was not determined. In light of the findings that unphosphorylated and phosphorylated CaD display quite different actin-binding properties [11,39] and intracellular distributions [16,21], variations in CaD phosphorylation may have contributed to this apparent discrepancy. To better understand the role of CaD phosphorylation in vascular SMCs, we have force-expressed phosphomimetic mutants of CaD in A7r5 cells, a model for remodelled or diseased vascular SMCs, and examined the resulting cells in terms of their morphology, migration activity, contractility and detachment kinetics. We focused on Erk and PAK by separately or simultaneously altering the residues of the respective modification sites, because both kinases have previously been shown to affect CaD's affinity for actin filaments. Our data indicated that in order for A7r5 cells to attain motility, phosphorylation of CaD by either kinase is an obligatory step, primarily through the modulation of the actin cytoskeleton dynamics, rather than the contractile machinery of the cell.

The phosphorylation-disabled CaD mutant, A1234, at both PAK- and Erk-sites hampered the migration activity to the greatest extent (to ~13% of the control cells; Fig. 2). Cell migration is a multiple-step process, which includes cell extension, attachment, contraction and rear detachment [29]. The observed slower migration could have resulted from one or more compromised steps in this process. However, when we examined the cell contractility by traction microscopy, we found that the A1234-expressing cells exhibited strongest traction force (Fig. 3B). Thus the overall cell motility must be dominated by step(s) other than contraction. Indeed, the detachment assay showed that A1234 mutant rendered A7r5 cells to round up and detach from the substratum more slowly than other variants (Fig. 5). Consistent with this observation is the fact that cells transfected with A1234 exhibited most robust stress fibers (Fig. 1). It is conceivable that these structures may be hard to disassemble. Together, these results suggest that it is the less dynamic actin cytoskeleton, which is overly stabilized by the unphosphorylatable CaD, that makes the cell more resistant to shape changes, and thereby hampering the cell motility. This interpretation agrees with Yamashiro's work [40], and reinforces our understanding about the functional role of CaD phosphorylation as a means to reverse the actin stabilizing effect of CaD.

The fact that Ser-to-Ala mutation at only four positions (amino acid residue-452, 482, 497 and 527) attains a reduction in cell motility of nearly 90% is quite remarkable. On one hand, it means phosphorylation at these four residues is *necessary *for cell migration, on the other hand, it also demonstrates that other mechanisms including phosphorylation at *other *positions only contribute no more than 13% of the overall migration activity. Previously, it has been suggested that Ca^2+^/calmodulin also regulates the CaD-imposed inhibitory effect [41]; our results argue that such an effect may only play a minor role in A7r5 cells, particularly in the absence of phosphorylation-mediated regulation. Transfection with A1A2 or A3A4 mutants of CaD inhibited approximately 50% of the extent by the A1234 mutant in cell migration when compared to the control cells (Fig. 2). This intermediate activity could be due to phosphorylation at residues that remain available. Indeed, Ser-527 (one of the Erk-sites) of the A1A2 mutant was found to be phosphorylated (Fig. 4). Thus it seems both MAPK and PAK contributed equally and additively to the overall motility of cultured aorta SMCs. The involvement of PAK, which is a downstream effector of Rac signaling, in cell migration is well established. That MAPK-mediated phosphorylation also enables cell migration is consistent with previous observations [14], and suggests the existence of cross-talks between MAPK- and PAK-pathways.

Force-expression of wild-type CaD (in wtCaD cells) decreased the motility of the A7r5 cells. This may be understood by the assumption that the total CaD level in these cells exceeds the capacity of the kinases and results in a net increase of unphosphorylated CaD, which causes inhibition of the cell motility. This situation is similar to that of the A1A2 and A3A4 cells, where phosphorylation of CaD is partially blocked. This interpretation is supported by the observation that these three types of cells exhibited about the same degree of suppression in motility. When we force-expressed D1234 mutant, we expected an increase in the motility, because the phospho-mimicking mutation might represent a scenario opposite to the fully inhibited state (e.g., A1234). Yet we found the D1234 mutant also suppressed the motility of the cells, although to a lesser extent (~24% inhibited). This could be attributed to the fact that such modification was irreversible. Not being able to be dephosphorylated, this CaD mutant disrupts the phosphorylation cycling of endogenous CaD and may thereby lead to inhibition of cell motility [19]. In the meantime, since D1234 mutant has a much weakened affinity for the actin filament, it cannot stabilize the cytoskeleton as much as A1234 or wt-CaD; so the D1234 mutant cells showed weaker stress fiber structure and similar contractility and detachment behaviors compared to the control cells.

Our data indicate that the migratory activity of cells changes in a reciprocal manner as the contractility of the cells, despite that contraction is an essential step during cell migration. The contractile activity, as evaluated by our assays based on the total strain energy the cell exerts on the substratum, requires active actomyosin interactions. However, an equally important factor is a sturdy cytoskeleton structure that provides a framework for such interactions and supports the contractility. Without firm actin cables, such as in the D1234-transfected cells, even a relatively high actomyosin ATPase activity would not be able to generate force. The cell contractility, the cytoskeleton stability, and the cell migration activity must therefore behave in a coordinated manner. On the other hand, the A1234-expressing cells, which produced highest traction force because of the stabilized actin cytoskeleton therein, must also have an actomyosin activity that is not severely inhibited. This is rather surprising in view of the overwhelming evidence that unphosphorylated CaD (i.e., A1234) inhibits such activity. One possible explanation is that the actomyosin interaction is activated through the binding of calmodulin to CaD, as the calmodulin-binding sites are still functional in these mutants. However, this mechanism apparently fails to restore the migration activity, since cells expressing A1234 were only 13% as motile as the control cells (Fig. 2). Clearly, the cytoskeleton stability is a more dominant factor than the actomyosin ATPase activity in the case of cell migration, because multiple steps (i.e., extension, attachment and detachment) in this process depend on the ability of actin filaments to be assembled and disassembled dynamically.

The finding that stabilization of the actin cytoskeleton by CaD is critical for cell motility provides the rationale for using CaD to curb SMC migration which in most cases is pathogenic. In particular, transfection of CaD in mice has been shown to suppress the growth of vascular SMCs and inhibit neointimal formation after angioplasty [42]. More recently it was reported that expression of CaD suppresses the invasive activity of cancer cells [43]. Our study further suggests that CaD with combined mutations at both PAK- and Erk-sites (e.g., A1234) may serve as a more effective therapeutic reagent in cancer and proliferative vascular diseases such as atherosclerosis and restenosis.

## Conclusions

In this study we showed that CaD suppresses the motility of vascular SMCs by stabilizing the actin filamentous structure. Despite intensive studies over the past three decades, the functional role of CaD in non-muscle cells remains elusive. In this regard, our data shed light onto the following aspects: (1) Phosphorylation of CaD at the Erk- and PAK-sites near the actin-binding sites of CaD is required for cell motility, because it reverses CaD's inhibitory effect and allows dynamic changes of the actin cytoskeleton. (2) Both Erk and PAK contribute equally and additively toward this regulatory role in cell migration and contraction. (3) Other mechanisms such as phosphorylation at residues other than the four positions (residues 452, 482, 497 and 527) or interaction with calmodulin only play a relatively minor role in modulating the motility of A7r5 cells. These new insights not only help us to better understand how CaD works, but also afford useful information on how cell motility is regulated. For SMCs, in particular, our findings suggest that mutations at CaD phosphorylation sites serve as a novel therapeutic strategy to combat vascular diseases such as atherosclerosis and restenosis.

## List of abbreviation used

CaD: caldesmon; Erk: extracellular signal-regulated kinase; FGF: fibroblast growth factor; h-CaD: heavy or smooth muscle-specific caldesmon; l-CaD: light or non-muscle caldesmon; MAPK: mitogen-activated protein kinase; PAK: p21-activated protein kinase; PBS: phosphate buffered saline; PFA: paraformaldehyde; SMC: smooth muscle cell.

## Competing interests

The authors declare that they have no competing interests.

## Authors' contributions

QJ carried out the cell biology studies, participated in the mechanical measurements and drafted the manuscript. RH carried out the immunoassays. SC participated in the design of the study. CLAW conceived of the study, and participated in its design and coordination of the experiments, and helped to write the manuscript. All authors read and approved the final manuscript.
